# Greatwall-Endos-PP2A/B55^Twins^ network regulates translation and stability of maternal transcripts in the *Drosophila* oocyte-to-embryo transition

**DOI:** 10.1098/rsob.240065

**Published:** 2024-06-19

**Authors:** Hélène Rangone, Laura Bond, Timothy T. Weil, David M. Glover

**Affiliations:** ^1^ Department of Genetics, University of Cambridge, Downing Street, Cambridge, UK; ^2^ Department of Zoology, University of Cambridge, Downing Street, Cambridge, UK; ^3^ Division of Biology and Biological Engineering, California Institute of Technology, 1200 E. California Blvd, Pasadena, CA 91125, USA

**Keywords:** Greatwall, Endosulfine, Pan Gu kinase, oocyte-to-embryo transition, maternal transcripts, *Drosophila*

## Abstract

The transition from oocyte to embryo requires translation of maternally provided transcripts that in *Drosophila* is activated by Pan Gu kinase to release a rapid succession of 13 mitotic cycles. Mitotic entry is promoted by several protein kinases that include Greatwall/Mastl, whose Endosulfine substrates antagonize Protein Phosphatase 2A (PP2A), facilitating mitotic Cyclin-dependent kinase 1/Cyclin B kinase activity. Here we show that hyperactive *greatwall^Scant^* can not only be suppressed by mutants in its Endos substrate but also by mutants in Pan Gu kinase subunits. Conversely, mutants in *me31B* or *trailer hitch,* which encode a complex that represses hundreds of maternal mRNAs, enhance *greatwall^Scant^*. Me31B and Trailer Hitch proteins, known substrates of Pan Gu kinase, copurify with Endos. This echoes findings that budding yeast Dhh1, orthologue of Me31B, associates with Igo1/2, orthologues of Endos and substrates of the Rim15, orthologue of Greatwall. *endos-*derived mutant embryos show reduced Me31B and elevated transcripts for the mitotic activators Cyclin B, Polo and Twine/Cdc25. Together, our findings demonstrate a previously unappreciated conservation of the Greatwall–Endosulfine pathway in regulating translational repressors and its interactions with the Pan Gu kinase pathway to regulate translation and/or stability of maternal mRNAs upon egg activation.

## 1. Introduction

In plant or animal embryos, transcripts and proteins deposited by the mother in the oocyte support the development of the newly created zygote before the onset of zygotic transcription [1–5]. In *Drosophila*, the Pan Gu kinase is activated at the oocyte-to-embryo transition and triggers a dramatic reorganization of the maternal translatome, on which depends the onset of the mitotic cycles [6–13].

Mitotic protein kinases regulate 13 initial successive nuclear division cycles in the *Drosophila* syncytial embryo. The activation of Cyclin-dependent kinase 1 (Cdk1)/Cyclin B triggers entry into mitosis, and its downregulation leads to mitotic exit [14,15]. Polo kinase promotes Cdk1 activity by phosphorylating its activating phosphatase Twine, the germline-specific homologue of Cdc25 in *Drosophila* [16,17]. Greatwall (Gwl) kinase enhances Cdk1 activity by antagonizing Protein Phosphatase 2A (PP2A), a major phosphatase counterbalancing Cdk1/Cyclin B activity [18–23]. Gwl, Mastl in mammals, phosphorylates proteins of the Endosulfine family to inhibit PP2A associated with a B55 regulatory subunit [24–27]. Inhibition of PP2A/B55 facilitates the activity of the Cdk1/Cyclin B complex required for mitotic entry and progression [28–31].

We identified the Gwl–Endos–PP2A pathway when investigating *Scant*, a gain-of-function allele of *Drosophila gwl* [26,28,32,33]. *Scant* encodes a hyperactive form of Gwl kinase that results in embryonic lethality when the maternal provision of the Polo kinase is reduced [32,33]. Our screen for suppressors of the maternal effect lethality of *polo gwl^Scant/++^* females identified revertants of the *gwl^Scant^* mutation that restore Gwl kinase activity to normal levels; duplications of the wild-type *polo* locus; and extragenic suppressors that include recessive mutations in *endos*, encoding Gwl’s principal Endosulfine substrate in *Drosophila* [26,33]. By contrast, mutations in *twins*, encoding the *Drosophila* B55 subunit of PP2A, enhance the sterility of the *polo gwl^Scant/++^* females [26,34]. During our initial screen for *polo gwl^Scant^* suppressors, our pilot studies also pointed towards possible restoration of female fertility with a mutant allele of *pan gu* (unpublished observations).

It has been previously shown that *endos* mutants have reduced levels of the M-phase regulators Polo and Twine in mature *Drosophila* oocytes, suggesting the possibility that Endos might exert post-transcriptional regulation of the levels of these proteins [35]. By contrast, Polo levels in cultured cells did not appear to be under Endos control [26]. This suggests that in the oocyte and syncytial embryo, Gwl and Endos might not only control the balance between Cdk1/CyclinB and PP2A/Twins but could also regulate the translation or stability of maternal mRNAs. Here, we describe genetic interactions between *gwl* and *pan gu (png*) subunits that support this view. Moreover, we show that Endos physically interacts with a protein complex that regulates translation at the oocyte-to-embryo transition. Together our study reveals the roles of the Gwl–Endos–PP2A pathway, shared with Png kinase in regulating the translation and/or stability of maternal mRNAs and in coordinating mitosis at the oocyte-to-embryo transition.

## 2. Results

### (a) Genetic interactions between the Gwl kinase and Pan Gu kinase pathways

**Figure 1.**
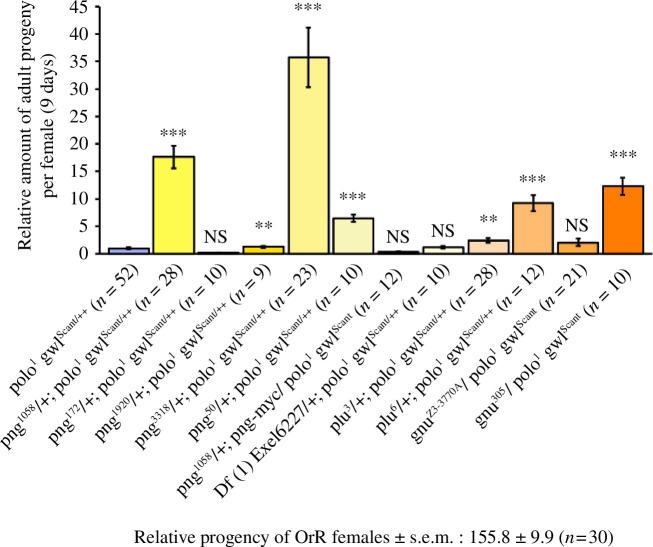
Suppression of the sterility of hyperactive Gwl kinase by mutations in Pan Gu kinase subunits. Females of genotype *polo^1^ gwl^Scant/++^* show strong maternal effect lethality measured here by the amount of adult progeny hatched after 9 days of egg laying and normalized to 1 for all experiments. The relative amount of progeny of mothers carrying specific mutant alleles of the indicated genes transheterozygous to *polo^1^ gwl^Scant^* is presented (error bars = s.e.m.). The relative number of progeny of wild-type OrR females is indicated. *n*, number of females analysed for each genotype. Statistical significance is by a Mann–Whitney *U*-test: **p < 0.01; ***p < 0.001; NS: non-significant.

A screen of mutant alleles of *png* revealed that they partially rescued the maternal effect lethality of *polo gwl^Scant/++^* females to varying extents (figure 1). To confirm the specificity of this rescue, we expressed a *png-myc* transgene in the background of *png^1058^*, an allele giving strong rescue, and found that this restored the maternal effect lethality of *polo^1^ gwl^Scant/++^* (figure 1). *png* encodes the catalytic subunit of protein kinase that is stoichiometrically associated with a regulatory subunit, Plutonium (Plu), and an activating subunit, Giant Nuclei (Gnu). Gnu acts on the Png–Plu subcomplex as the oocyte passes through the oviduct, triggering a peak of Png kinase activity in the early syncytial embryo [6,8,13]. Mutations in any of these three genes lead to a failure to arrest development after completion of meiosis and the formation of giant nuclei in the embryo through repeated rounds of S-phase in the absence of mitosis [36–38]. We therefore asked whether mutations in either *plu* or *gnu* could also suppress the maternal effect lethality of *polo^1^ gwl^Scant/++^* females and found allele-specific rescue of *polo^1^ gwl^Scant/++^*-derived embryo development (figure 1). The nature of the allele specificity of the rescue by *png*, *plu* or *gnu* alleles that give this rescue indicates that the genetic interaction is independent of the catalytic activity of Png. All the *png* alleles tested induced a decrease in the level of Cyclin B in syncytial embryo extracts (electronic supplementary material, figure S1), an expected phenotype in embryos laid by *png* defective females supporting their efficiency [6,7,10]. However, the extent of the rescue does not correlate with the previously described strength of the *png* alleles [6], and a deficiency uncovering *png* did not restore any embryonic viability (figure 1), indicating that Png protein is physically required for the rescue independently of kinase activity. Thus, it is possible that mutations of Png kinase subunits affecting the structural integrity of the protein kinase complex (and possibly its physical interactions) can rescue the maternal effect lethality of the Gwl^Scant^ hyperactive protein kinase (see §3).

### (b) The Gwl and Pan Gu pathways intersect through PP2A/B55^Twins^

**Figure 2.**
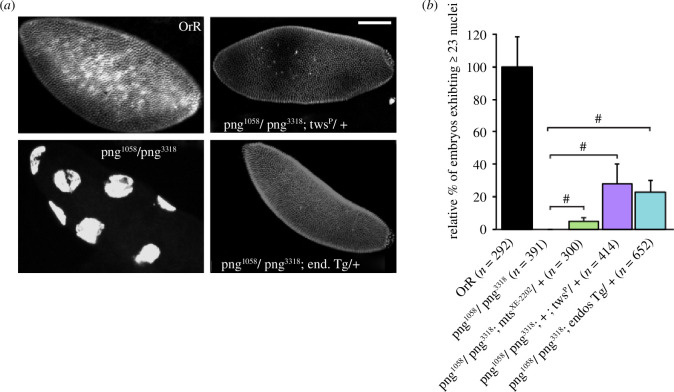
Suppression of *pan gu* by mutations reducing the activity of PP2A. (*a*) Propidium iodide stained syncytial embryos from OrR wild-type mothers or from heterozygous *png* mothers, in the absence of other mutations or additionally hemizygous for a mutation in *tws,* encoding the 55 kDa regulatory subunit of PP2A, or carrying one copy of an *endos* transgene. Scale bar, 100 μm. (*b*) Histogram represents quantification of embryos with 23 or more nuclei relative to wild-type (OrR) and expressed relative to the expected proportion of mothers hemizygous for the *mts* (encoding the catalytic subunit of PP2A) or *tws* mutation or the *endos* transgene (error bars = s.e.m.). *n*, number of syncytial embryos analysed in at least three independent experiments. We have never observed embryos with greater than 23 nuclei arising from *png* mothers (#, phenotype observed in indicated genotypes versus *png-*derived embryos).

As Gwl is required for the correct regulation of the mitotic cycle and Png for the activation of the mitotic cycles in the syncytial embryo, we considered that the pathways may share at least one common regulatory element. Of several possible mitotic regulators, it has been reported that the *png* mitotic phenotype can be rescued by mutation of the catalytic subunit of PP2A [7,9], encoded by *microtubule star* in *Drosophila*. PP2A is a trimeric complex that, in addition to its catalytic subunit (C), has a structural subunit (A) and one of several possible regulatory subunits (B). As Endos that has been phosphorylated by Gwl specifically inhibits the PP2A complex containing the B55 regulatory subunit (encoded by *twins* in *Drosophila*), this raised the question of whether downregulation of PP2A/B55^Twins^ would enable some rescue of the *png* mutant phenotype. We approached this in two ways: (i) by reducing the function of PP2A/Twins with a *microtubule star* (*mts*) or a *twins* (*tws*) mutant allele (the catalytic and regulatory subunits, respectively, of the phosphatase complex) and (ii) by expressing a transgene of *endos* to increase the inhibition of PP2A/Twins (figure 2). Embryos derived from *png^1058^/png^3318^* females undergo no more than four cycles of mitosis before their nuclei embark upon endoreduplication. Neither we nor, to our knowledge, others studying *png* [38] have seen the development of a greater number of nuclei in embryos derived from *png^1058^/png^3318^* females. In both the above cases, however, a proportion of embryos derived from *png^1058^/png^3318^* females with compromised PP2A activity developed through considerably more mitotic cycles to have hundreds of nuclei (figure 2*a*). Thus, our findings extend previous work indicating that the *png* mitotic phenotype can be rescued by mutation of the catalytic subunit of PP2A [7,9]. Thus, reducing PP2A/B55^Twins^ protein phosphatase activity appears sufficient to overcome the block to mitotic progression imposed by this *png* mutant background (figure 2 and electronic supplementary material, table S1). These results suggest that the Gwl–Endos and Png pathways can synergize to enhance Cdk1 activity in a manner that accords with the ability of Gwl to downregulate PP2A activity through Endos.

### (c) The Gwl and Pan Gu pathways intersect through the regulation of maternal transcript translation and stability

Upon activation, the *Drosophila* egg is triggered to undertake its repeated mitotic cycles by a brief window of Png kinase activity that results in hundreds of maternal mRNAs being repressed and hundreds more being activated [12]. Png promotes the translation of Smaug, a translational repressor that promotes de-adenylation of a set of mRNAs corresponding to the majority of the repressed transcripts [11,39]. Png also phosphorylates Trailer Hitch (Tral) suppressing its ability to repress mRNA translation [40]. Tral forms a complex together with Me31B, a DEAD box helicase, and Cup, an eIF4E-binding protein [41–43]. The binding of Cup to eIF4E allows the Cup–Tral–Me31B complex to disrupt the eIF4E–eIF4G interaction and inhibits the initiation of translation [41,44–46]. Me31B is also a Png substrate [40].

**Figure 3.**
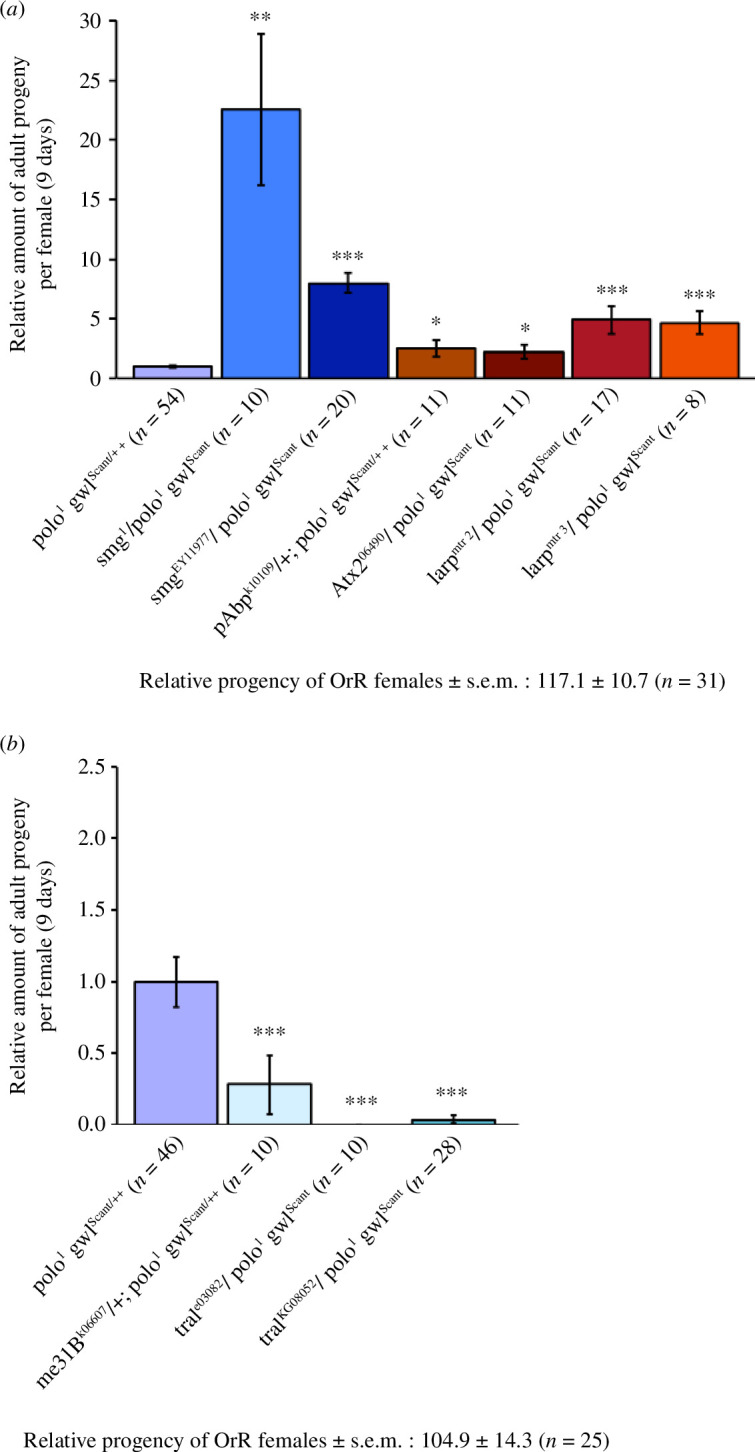
Genetic interactions between *greatwall^Scant^* and genes that regulate translation and stability of maternal mRNAs. Suppression (*a*) and enhancement (*b*) of the sterility of *polo^1^ gwl^Scant/++^* females. Histograms represent the relative amount of adult progeny hatched after 9 days of egg laying by mothers carrying the indicated mutant allele transheterozygous to *polo^1^ gwl^Scant^* (error bars = s.e.m.). The relative number of progeny of wild-type OrR females is indicated. *n*, number of females analysed for each genotype. Statistical significance is by a Mann–Whitney *U*-test: *p < 0.05; **p < 0.01; ***p < 0.001; NS: non-significant.

Since mutations in Png suppressed the maternal effect lethality of *polo^1^ gwl^Scant/++^*, we asked whether the *png* downstream effectors mentioned above would also show this ability. We first investigated mutations in Smaug and found that, indeed, *smaug* mutants showed similar levels of suppression of maternal effect lethality as mutants in Png and its subunits (figure 3*a*). We then asked whether mutants in *me31B*, *tral* or *cup* would show any genetic interaction with *polo^1^ gwl^Scant/++^*. Mutations in *cup* had no significant effect upon the numbers of progeny arising from *polo^1^ gwl^Scant/++^* mothers (not shown), whereas in contrast to mutations in *smaug*, we found that mutations in *tral* or *me31B* all tended to enhance the *polo^1^ gwl^Scant/++^* phenotype (figure 3*b*). Interestingly, we also noticed partial rescue by alleles of *poly(A) binding protein* (*pAbp*), *Ataxin 2* (*Atx2*) and *La-related protein* (*larp*) (figure 3*a*), in line with a shared function of these proteins in promoting translation in diverse cellular contexts [47–50]. These opposing effects on the *polo^1^ gwl^Scant^* sterility would correlate with the respective repressor and activator functions of translation attributed to the Tral–Me31B and pAbp–Atx2 complexes (figure 3).

Thus, the restriction of embryonic development by hyperactive Gwl kinase in the presence of diminished Polo kinase is suppressed not only by mutations in *endos*, encoding Gwl’s major mitotic substrate but also by mutations in genes regulating the translation of maternal mRNAs. These include allele-specific suppression effects of mutations in *png*; mutations in *smaug*, a gene required for the degradation of maternal transcripts of cell cycle regulatory proteins; and mutations in members of the Larp–pAbp protein complex implicated in translational regulation of cell cycle regulatory genes in the syncytial embryo [50]. In contrast, genes that are negatively regulated by Png appear to act as enhancers.

### (d) Endos physically interacts with protein complexes involved in regulation of maternal transcripts and proteins

The abovementioned experiments suggested an involvement of the Gwl–Endos pathway in the regulation of the stability or translation of maternal transcripts, either through direct interactions or as secondary consequence of its regulation of PP2A activity. To evaluate these possibilities, we chose to identify protein partners of Endos by immunoprecipitation of the protein from embryo extracts followed by mass spectrometry. We carried out eight such pulldowns and found that Endos repeatedly co-purified with eight proteins in all cases (table 1 and electronic supplementary material, table S2). The presence or absence of phosphatase inhibitors had no clear consequence for the identity of these repeatedly co-purifying proteins. One of these proteins was the ubiquitin E3 ligase, Early Girl, previously described as an Endos partner [27,35]. The three members of the Tral–Me31B–Cup complex also co-purified with Endos as did eIF4E and pAbp (table 1). This is in line with previous reports that the Me31B–Cup–Tral complex together with eIF4E and pAbp are associated with the majority of transcripts in the *Drosophila* embryo [43]. We also repeatedly co-purified Lost (also known as Growl) and Ypsilon Schachtel (table 1), proteins that localize to P bodies, granules facilitating the storage of RNA and translational regulation [51–53]. The proteins that co-purified with Endos were only occasionally immunoprecipitated (and never as a whole complex) with antibodies against Cyclin B, Cp110 or D-CLIP190, highlighting the specificity of the interactions with Endos (electronic supplementary material, table S3). mRNA regulatory proteins were not pulled down with Endos from cultured *Drosophila* cells (data not shown), indicating that the association we describe here reflects the regulation of maternal transcript stability and translation in the early embryo.

**Table 1.** Physical interactions between Endos and proteins that regulate translation and stability of maternal mRNAs.

Protein name (CG number)	Exp 1	Exp 2	Exp 3	Exp 2 Froz.	Exp 2 Buf.	Exp2 + PPase Inh.	Exp3 + PPase Inh.	Exp2 Buf. + PPase Inh.
Endosulfine (CG6513)	344.0 (95.0)	5075.0 (0.0)	2311.0 (0.0)	4090.0 (0.0)	2782.0 (0.0)	5412.0 (0.0)	2002.0 (144.0)	3497.0 (0.0)
Me31B (CG4916)	472.0 (0.0)	1297.0 (39.0)	49.0 (141.0)	2176.0 (0.0)	933.0 (0.0)	665.0 (39.0)	396.0 (266.0)	1102.0 (0.0)
Trailer hitch (CG10686)	1070.0 (944.0)	2112.0 (1136.0)	461.0 (0.0)	3831.0 (0.0)	289.0 (0.0)	3788.0 (1136.0)	1001.0 (684.0)	789.0 (0.0)
Cup (CG11181)	1058.0 (117.0)	2060.0 (72.0)	234.0 (176.0)	1713.0 (0.0)	131.0 (0.0)	1647.0 (72.0)	515.0 (38.0)	387.0 (0.0)
PolyA-binding protein (CG5119)	518.0 (45.0)	1635.0 (0.0)	242.0 (76.0)	1382.0 (0.0)	913.0 (0.0)	1417.0 (0.0)	332.0 (169.0)	1214.0 (0.0)
eIF4E1 (CG4035)	119.0 (77.0)	179.0 (0.0)	118.0 (0.0)	206.0 (0.0)	178.0 (0.0)	349.0 (0.0)	112.0 (0.0)	198.0 (0.0)
Lost (CG14648)	343.0 (0.0)	829.0 (0.0)	135.0 (69.0)	563.0 (0.0)	341.0 (0.0)	372.0 (0.0)	148.0 (55.0)	126.0 (0.0)
Ypsilon schachtel (CG5654)	222.0 (0.0)	463.0 (0.0)	34.0 (0.0)	312.0 (0.0)	284.0 (0.0)	287.0 (0.0)	194.0 (0.0)	286.0 (0.0)
Early girl (CG17033)	297.0 (0.0)	1113.0 (0.0)	388.0 (0.0)	1106.0 (0.0)	539.0 (0.0)	700.0 (0.0)	307.0 (0.0)	1384.0 (0.0)

Mass spectrometric analysis of proteins immunoprecipitated from embryo extracts by an anti-Endos antibody. Endos interacting proteins were identified in three independent experiments (Exp 1, 2 and 3) further testing the effects of immunoprecipitation from frozen embryos samples (Exp 2 Froz.); immunoprecipitation in presence of higher detergent concentration (Exp 2 Buf.); following addition of protein phosphatases inhibitors (+ PPase Inh.).

Table shows Mascot scores of proteins with control pulldown scores in brackets (full dataset provided in electronic supplementary material, table S2).

A full list of other proteins that co-purify with Endos from *Drosophila* embryos is given in electronic supplementary material, table S2. A classification of GO terms, identifying biological processes and cellular components associated with these proteins, suggests the possibility that in addition to interacting with proteins that regulate mRNA translation and stability, Endos might also associate with proteins that function in the splicing of mRNA (electronic supplementary material, figure S2). However, it should be noted that, in contrast to the eight proteins regulating translation and mRNA stability indicated above, splicing proteins were not present in all of the Endos pulldowns. It will be of considerable future interest to further study such interactions and whether they might be phospho-dependent.

The interaction of Endos with Me31B is in line with previous studies in budding yeast, which have shown Rim15, counterpart of Gwl, to phosphorylate the Endos orthologues Igo1 and Igo2. Once phosphorylated, Igo1/2 associates with the mRNA decapping activator Dhh1, orthologue of Me31B [54], preventing its activity. In yeast, this is a response to TORC1 and PKA signalling following starvation and has the consequence of protecting mRNAs from degradation via the 5′–3′ mRNA decay pathway to initiate the G0 program.

### (e) Endos has multiple roles in regulating levels of mitotic regulators

**Figure 4.**
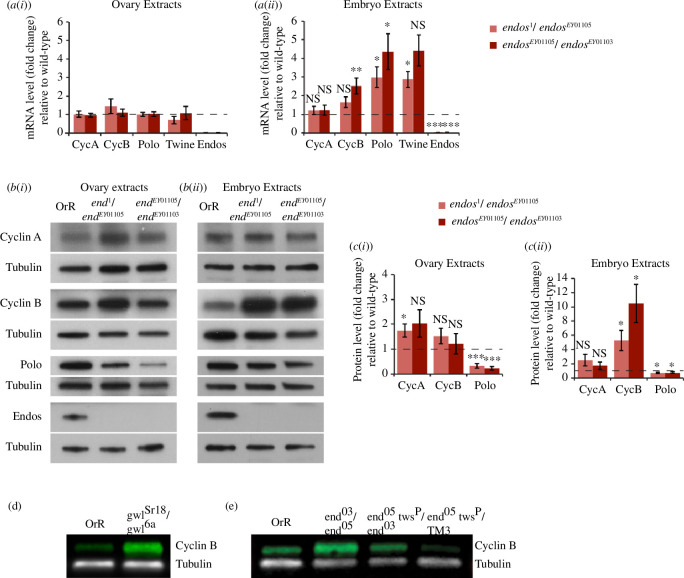
Endos regulates levels of multiple maternal transcripts and proteins. (*a*) Quantification of indicated transcripts by qRT–PCR in ovary (i) or 0–2 h embryo extracts (ii), showing transcript levels in tissue or embryos derived from mothers heterozygous for the two indicated *endos* mutant alleles normalized to wild-type (OrR) levels set to 1 (error bars indicate s.e.m.). Endos regulates transcript levels in syncytial embryos but not ovaries. Significance by one-sample *t*‐test *p < 0.05; **p < 0.01; ***p < 0.001 as indicated. NS indicates non-significant. Transcript levels are average of at least four experiments from four independent samples for ovary extracts and at least six experiments from five independent samples for embryo extracts. (*b*) Endogenous protein levels corresponding to selected transcripts analysed in (*a*) assessed by western-blot analysis of ovary (i) or 0–2 h embryo extracts (ii). Tubulin provides a reference control. (*c*) Quantifications of western blots as in (*b*) indicate Endos regulates the levels of some proteins in ovaries (i) and in embryos (ii) extracts. The relative amount of proteins in indicated *endos* transheterozygous normalized to wild-type (OrR) levels set to 1 (error bars = s.e.m.). Statistical significance analysed by a one-sample *t*‐test: *p < 0.05; **p < 0.01; ***p < 0.001; NS: non-significant. Protein levels are average from at least seven experiments from three independent samples for ovary extracts and from at least five experiments from four independent samples for embryo extracts. (*d*) Endogenous levels of Cyclin B, analysed by western blot in extracts from 0 to 2 h syncytial embryos laid by *gwl^Sr18^/gwl^6a^* mothers, are regulated by Gwl. Tubulin provides a reference control. Four independent sets of samples were prepared. A representative image is shown. (*e*) Endogenous protein level of Cyclin B assessed by western-blot analysis in 0–1 h embryo extracts with tubulin as a reference control. Four independent sets of samples were prepared from embryos laid by mothers of indicated genotypes. A representative image is presented.

*Drosophila* Me31B is required for translational silencing during the transport of mRNA to the oocyte [55,56]. In the early embryo, it can either repress the translation of maternal transcripts or promote their degradation [41,43,57–59]. As the translational regulation of cell cycle regulatory proteins is a critical aspect of early *Drosophila* embryogenesis, we chose to analyse levels of a set of mitotic regulators anticipated to be under the control of Endos [26,35,60]. We found that transcript levels of Cyclin A, Cyclin B, Polo and Twine were not significantly different in wild-type versus *endos* mutant ovaries (in two different combinations of *endos* mutant alleles, *endos* ^*1*^/*endos* ^*EY01105*^ or *endos* ^*EY01105*^/*endos* ^*EY01103*^). However, there was an increase in Cyclin B, Polo and Twine transcripts in *endos*-derived mutant embryos (figure 4*a*). As no transcription is yet taking place in the early syncytial embryo, we attribute the higher levels of transcripts as a consequence of their reduced degradation. This suggests that Endos would normally act to destabilize these maternal transcripts in the embryo.

We then assessed the levels of the proteins corresponding to these transcripts in ovary and embryo extracts by western-blot analysis (figure 4*b,c*). In the ovaries of *endos* mutant females, we observed the previously reported decrease of Polo levels [26,35] but otherwise little change in levels of Cyclins A and B. In contrast, the level of Cyclin B protein was greatly enhanced in *endos-*derived embryos, a finding consistent with the delays in mitotic exit that arise as a consequence of loss of activity of the Gwl–Endos pathway. Accordingly, *gwl* mutations also led to an increase in Cyclin B levels, as also occurs in mitotically delayed cells following Gwl depletion [18,33] (figure 4*d*).

We then asked whether the increase in Cyclin B levels reflected the lack of the inhibitory effect of Endos upon PP2A. We did this by making mothers carrying two mutant copies of *endos* and a single mutant copy of *tws*, encoding the B55 regulatory subunit of PP2A (figure 4*e*). We found that reducing PP2A activity in the *endos* mutant background in this way restored Cyclin B towards wild-type levels. Thus, the effects of *endos* mutations upon Cyclin B levels accord with the known role of Endos in regulating PP2A activity through inhibition of the B55 regulatory subunit [24–27].

### (f) Endos regulates P-body morphology

The association of Endos with Me31B, a marker of embryo P bodies in embryos, which serve as centres for RNA metabolism [51,52], led us to examine the distribution of Endos in syncytial embryos of a line expressing GFP-tagged Me31B. We found that the two proteins were present throughout the cytoplasm as punctate bodies, only a very small proportion of which were associated with both proteins (electronic supplementary material, figure S3*a*). We found similar results when Me31B was localized by immunostaining, with Endos being present only in a sub-set of Me31B stained P bodies and with the two proteins exhibiting different nuclear localizations; while Me31B was excluded from the nucleus, Endos was not (electronic supplementary material, figure S3*b*). The specificity of the cytoplasmic punctate distribution of Endos was confirmed by its strong reduction in embryos derived from *endos^EY01105^/endos^EY01103^* females or *endos^1^/endos^EY01105^* females (electronic supplementary material, figure S3*a*). Noticeably, Me31B::GFP foci appeared larger in *endos*-derived mutant embryos, suggesting that Endos might regulate Me31B’s distribution (electronic supplementary material, figure S3*a*).

**Figure 5.**
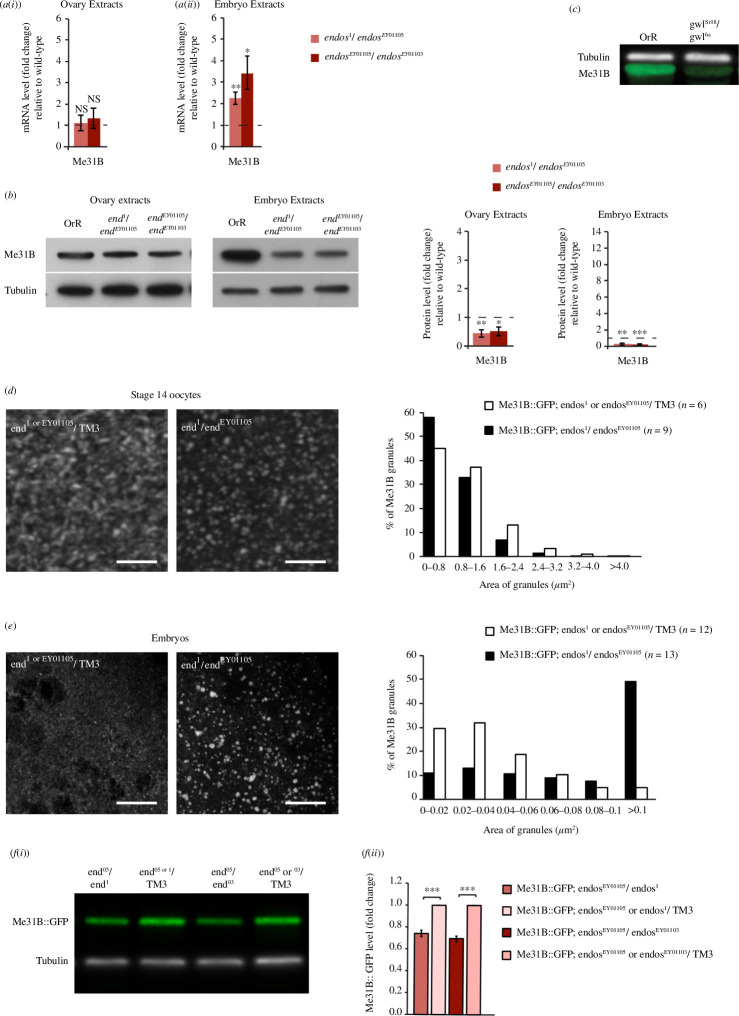
Endos regulates the levels of Me31B and its distribution. (*a*) Quantification of Me31B transcripts by qRT–PCR in ovary (i) or 0–2 h embryo extracts (ii) in tissue or embryos derived from mothers heterozygous for the two indicated *endos* mutant alleles normalized to wild-type (OrR) levels set to 1 (error bars indicate s.e.m.). Significance by one-sample *t*‐test: *p < 0.05; **p < 0.01 as indicated. NS indicates non-significant. Transcript levels are average of at least three experiments from three independent samples for ovary extracts and at least six experiments from five independent samples for embryo extracts. (*b*) Endogenous Me31B levels assessed by western-blot analysis of ovary or 0–2 h embryo extracts. Tubulin provides a reference control. Quantifications of Me31B level and statistical significance by a one-sample *t*‐test: *p < 0.05; **p < 0.01; ***p < 0.001. Protein levels are average from at least eight experiments from six independent samples for ovary extracts and from at least eight experiments from five independent samples for embryo extracts. (*c*) Endogenous levels of Me31B, analysed by western blot in extracts from 0 to 2 h syncytial embryos laid by *gwl^Sr18^/gwl^6a^* mothers, are regulated by Gwl. Tubulin provides a reference control. Four independent sets of samples were prepared. A representative image is shown. (*d*,*e*) Distribution of Me31B::GFP in stage 14 oocytes (*d*), and 0–2 h embryos (*e*) derived from females expressing Me31B::GFP additionally carrying the indicated *endos* alleles with *endos/TM3* balancer as control. Representative images are shown (scale bar = 10 μm). Graphs show the area of individual granules. Three fully independent experiments were performed. The number (*n*) of oocytes or embryos analysed for each genotype is indicated. For simplicity, the set of data of one *endos* mutant combination is presented, and the second *endos* mutant combination exhibits similar distributions. (*f*) Western-blot analysis of the level of Me31B::GFP in 0–1 h syncytial embryo extracts from females expressing Me31B::GFP additionally carrying the indicated *endos* alleles with *endos/TM3* balancer as control (i). Tubulin provides a reference control. Quantifications of western blots (ii) indicate the level of Me31B::GFP in *endos* mutant derived embryos relative to the amount in the matching control over balancer set to 1 (error bars = s.e.m.). Statistical significance is by a one-sample *t*‐test: ***p <0.001. Protein levels are average from three experiments with four independent samples.

To further investigate the role of Endos in regulating the expression of maternal proteins, we tested whether Me31B::GFP distribution was affected in oogenesis by the abovementioned two combinations of mutant *endos* alleles. Me31B is gradually expressed from the earliest stages of oogenesis, accumulates in granules in the mature oocyte and disperses in the embryo following the egg activation [51,55]. We observed no significant effect of the *endos* mutations on levels of Me31B transcripts in ovarian extracts, but the levels of protein were reduced (figure 5*a,b*). However, *Me31B* transcript levels were elevated in *endos-*derived embryos, and levels of Me31B protein were diminished (figure 5*a,b*). Moreover, the amount of Me31B protein was also strongly reduced in *gwl* mutant embryos in line with a requirement for Gwl-mediated phosphorylation of the Endos protein to maintain Me31B levels (figure 5*c*). While Me31B::GFP followed a similar distribution between diffuse and punctate staining in the *endos* mutants as in controls throughout oogenesis (data not shown; for stage 14 oocyte, see figure 5*d*), punctate staining was less apparent in control embryos where Me31B-containing granules appeared smaller than in *endos-*derived embryos (figure 5*e*). Consistent with the levels of the endogenous Me31B protein in the *endos*-derived embryos, the total levels of Me31B::GFP in the *endos*-derived embryos were also diminished (figure 5*c*). Thus, Gwl-phosphorylated Endos appears to be required, directly or indirectly, for the stability of Me31B in embryos following their activation and in the absence of Endos, the remaining Me31B coalesces into P bodies. Collectively, this supports a direct or indirect role for *endos* to destabilize Me31B transcripts while ensuring their translation in early embryogenesis, processes that would be associated with the diminution of Me31B granules during the oocyte-to-embryo transition.

## 3. Discussion

**Figure 6.**
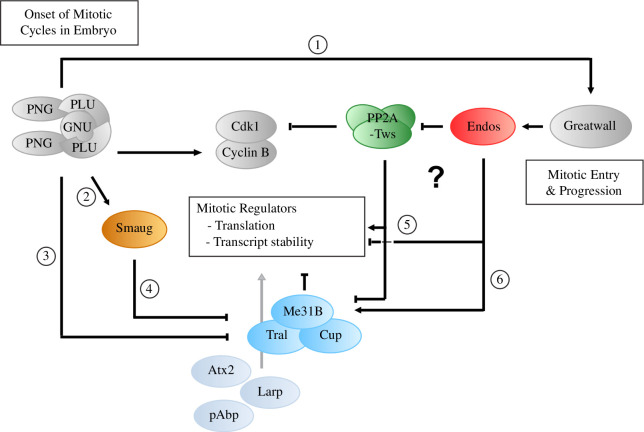
Pan Gu and Gwl pathways cooperate to enhance Cdk1/Cyclin B activity during the early embryonic divisions. Schematic to integrate interactions between the pathways from previously reported findings and from the current study. Png kinase regulates the stability and translation of maternal mRNAs in the transition from oocyte to embryo development including stimulation of Cyclin B translation. Gwl kinase phosphorylates Endos allowing it to antagonize PP2A/B55^Twins^, thus favouring Cdk1 activity. The lethality of *polo^1^gwl^Scant/++^* embryos reflects the hyperactivity of Gwl kinase encoded by this allele. Thus, mutations that downregulate Gwl or the mitotic pathways promoted by Gwl would suppress the *polo^1^ gwl^Scant^* phenotype. Mutations in Png kinase or Smaug would suppress because Png is required for *gwl* translation at egg activation (arrow 1) [12] and Smaug, whose translation is promoted by Png at egg activation (arrow 2) [11,12], acts together with Png to promote expression of mitotic regulators critical for the Gwl–Endos pathway. This is achieved because Png phosphorylates Me31B and Tral thereby inactivating these translational repressors (arrow 3*)* [40], and Smaug can also repress Tral, Cup and Me31B transcripts (arrow 4*)* [61]. This concurs with the ability of *tral* and *me31B* mutant alleles to enhance the sterility of the *polo^1^ gwl^Scant^* ^/++^ females and the ability of mutations in *atx2*, *pAbp* and *larp* to suppress this sterility (figure 3). Endos also acts to destabilize maternal transcripts for mitotic regulators, which are consequently elevated in *endos* hypomorphic mutants (arrow 5; figure 4). This could possibly be mediated, in part, through Endos’ physical association with the Me31B, Tral, Cup complex (arrow 6; table 1) and the remodelling of the P bodies (figure 5). However, the Endos requirement for translation appears specific to the mRNA. The mRNAs for several mitotic regulators are increased in *endos* hypomorphs, whereas affects upon the protein level vary in *endos* or *gwl* hypomorphs. Notably, levels of Me31B are reduced, although residual Me31B shows a more prominent association with P bodies. The effects of Endos could, at least partially, be mediated through its downstream target PP2A/B55^Twins^ (arrows 5, 6, ?) as suggested by the antagonism on Cyclin B levels.

Together, the abovementioned findings suggest a role for the Gwl kinase substrate, Endos, in the nuclear division cycles in the syncytial *Drosophila* embryo both by regulating mitotic progression and the translation and stability of mRNA. Such a role for the Gwl kinase complements that of the Png kinase; the former acts to promote mitotic entry and progression, and the latter to ensure the onset of mitotic cycles at the onset of zygotic development; both pathways influence the translation of maternal mRNAs (figure 6). This interaction between the two protein kinases ensures successful mitotic cycles in the early embryo. Png promotes the synthesis of Cyclin B and Smaug [10–12]. Gwl kinase phosphorylates Endos allowing it to antagonize PP2A/B55^Twins^, thus favouring Cdk1 activity [26,27]. How might mutants for *png*, *plu*, *gnu* or *smaug* suppress the *gwl^Scant^ polo* maternal effect? The *gwl^Scant^* allele encodes a hyperactive kinase that leads to lethality in the syncytial embryo in the presence of reduced levels of the Polo mitotic kinase. *gwl^Scant^ polo* is known to be suppressed by mutations that downregulate Gwl or Endos or that enhance Polo expression [26,33]. Thus, Png kinase or Smaug mutants would suppress *gwl^Scant^ polo* if they resulted in downregulation of the hyperactive Gwl^Scant^ kinase, its Endos substrate or the pathways that Gwl:Endos positively regulate. This could be achieved through the downregulation of mitotic pathways promoted by Png and Smaug, which together inactivate the translational repressor complex of Me31B, Cup and Tral, leading to the expression of many maternal mRNAs including those for mitotic regulators. It could be achieved through the requirement for Png to translationally activate Gwl mRNA at egg activation [11,12]. An alternative is that the reduced level of Cyclin B and hence Cdk1 activity in *png* mutants rebalances the elevated Gwl^Scant^ kinase activity in accordance with the documented role of Png on Cyclin B expression [6,7,10]. However, on its own, it could not account for the suppression of *gwl^Scant^ polo* since the *png^172^* allele or the *png* deficiency reduce Cyclin B levels in embryos but do not restore female fertility.

Elevated expression of Endos or to some extent mutation in *tws*, encoding the B55 regulatory subunit of PP2A, also suppress *png* mutants. We interpret this suppression of the *png* mitotic phenotype as a consequence of increased activation of the Cdk1/Cyclin B mitotic kinase or its substrates and promotion of mitosis by elevated Endos and decreased PP2A/B55^Twins^ activity echoing a previous finding of partial rescue of *png* by *mts* mutants [7,9]. Indeed, this was previously interpreted as the promotion of the mitotic state through the global loss of PP2A function. However, the precise regulatory events may be more complex because phosphorylation of Gnu by Cdk1/Cyclin B prevents Gnu’s association with, and thereby prevents activation of, the Png kinase [13]. If the protein phosphatase opposing this inhibitory phosphorylation is PP2A/Twins, then PP2A/Twins could potentially both activate the Png complex to promote the onset of the mitotic cycles and oppose Cdk1/Cyclin B favouring mitotic exit. Such diverse functions may operate at different stages of the oocyte-to-embryo transition.

The allele specificity of *png*’s suppression of *polo^1^ gwl^Scant^* indicates an independence of Png kinase activity. The mutant forms that give the weakest or no suppression of the *polo^1^ gwl^Scant^/++* phenotype lie in the catalytic loop (allele 1920, residue 140) or DFG domain (allele 172, residue 157), respectively, and so at the interface between the two domains of the kinase important for catalytic activity. The mutants showing the strongest suppression of *polo^1^ gwl^Scant/++^* lie either near the N-terminus (allele 3318, residue 17) or the C-terminus (allele 50, residue 250; allele 1058, residue 265) of this 291 amino acid molecule. This suggests that these N- and C-terminal regions may affect physical interactions between Png and its two partner proteins and/or with other molecules, whereas mutations in the core of the kinase would be less prone to disturb such interactions (e.g. allele 172). The consequences of these N- and C-terminally located mutations upon interactions of the catalytic subunit with its regulatory subunits has, to our knowledge, not been examined. However, in this light, we note that Gnu physically interacts with RNP granules and has been proposed to bring Png to its initial targets, translational repressors in RNP granules [62]. The interactions of the Png subunits and their targeting to substrates may thus play important roles in the spatio-temporal regulation of translational activation.

Png exerts control over maternal mRNAs by phosphorylating Me31B and Tral, thereby inactivating the translational repressor activity in the case of Tral [40]. Early in development, Me31B represses the translation of maternal mRNAs, whereas later, its levels diminish in a manner dependent upon Png kinase and the remaining Me31B promotes mRNA destruction [43]. Smaug can also repress Tral, Cup and Me31B transcripts [61]. These functions concur with the ability of *tral* and *me31B* mutant alleles to enhance the sterility of the *polo^1^ gwl^Scant^* ^/++^ females. The opposing ability of mutations in *atx2*, *pAbp* and *larp* to suppress this sterility accords with the shared functions of these proteins in promoting translation [47–50]. Thus the respective functions of the Tral–Me31B and pAbp–Atx2 complexes in repressing and activating translation are seen in opposite effects upon *polo^1^ gwl^Scant^* sterility.

Endos appears to increase the stability of Me31B, and it also physically associates with the Me31B, Tral and Cup complex in early embryos. We have not been able to observe such an association when precipitating Endos from extracts of cultured cells (data not shown). Thus the association we observe could reflect a specific need to destabilize maternal transcripts for mitotic regulators, which are elevated in *endos* hypomorphic mutants. The association of Endos with Me31B appears to have been conserved as such an association has been reported in budding yeast between the Endos orthologues, Igo1/2, and the Me31B orthologue, Dhh1 [54]. However, phosphorylation of Igo1/2 by the Gwl orthologue, Rim15, facilitates association of the Igo proteins with Dhh1 to shelter newly expressed mRNAs from degradation and enabling their translation during initiation of the G(0) program. In contrast, transcript levels for mitotic regulators show an increase in *endos* mutant embryos, suggesting that Endos has the opposite role in *Drosophila* to destabilize transcripts. That the Endos and Igo1/2 orthologues have opposing roles in flies and budding yeast is also seen in the requirement for the pathway in mitotic activation. In *Drosophila*, as in *Xenopus* and human cells, the Gwl–Endos pathway may be viewed as a mitotic activator as a result of its inhibition of PP2A/B55 [21,24–27,30]. In budding yeast, however, Cdc14 is the main Cdk1-antagonizing phosphatase and PP2A/Cdc55 phosphatase promotes mitotic entry. In line with this reversed requirement for PP2A in mitosis, Igo1/2, phosphorylated by Rim15, binds to PP2A/Cdc55 to act as positive regulators of PP2A in budding yeast [29]. The finding that Endos and Igo1/2 have opposite roles in regulating mRNA stability and in regulating mitotic entry in flies and budding yeast suggests some commonality to molecular mechanism. It raises the possibility that PP2A/B55 may play a role in regulating Endos or Igo1/2 functions at Me31B or Dhh1, respectively, in the two organisms, even though the phosphatase is not an obvious component of the decapping complex.

In *Drosophila*, it would be possible that the role of Endos in regulating translation and/or mRNA stability could be regulated through localized inhibition of PP2A/B55^Twins^ at the Me31B foci. However, Endos does not appear strongly enriched at these foci, suggesting that such regulation could also occur at distant sites. Cytoplasmic Endos is specifically required to interact and inhibit PP2A/B55^Twins^ in the cytoplasm of the syncytial embryo upon Gwl nuclear export on mitotic entry and has been suggested to exhibit features of a phase-separating protein [63]. Phase separation properties are also attributed to Me31B, which would shape the physical properties of the P bodies in the oocyte and support changes in their composition and distribution in the embryo after egg activation [64]. Pinpointing the precise spatio-temporal interactions of Endos with Tws, Me31B or other P-body partners, such as Tral, remains a future challenge to decipher the regulation of maternal transcripts and proteins downstream of the Gwl–Endos pathway.

Although mutations in *endos* have similar effects in elevating Cyclin B and Me31B transcripts in the embryo, we note that the consequences on protein levels are quite different. In part, this can be owing to the large variation in protein levels in response to a combination of translational and post-translational control as uncovered by Kronja *et al*. [12]. Indeed, Cyclin B protein levels follow the expected requirements for Gwl–Endos in mitotic progression; mutation in *gwl* or *endos*, which leads to mitotic delay, results in elevated Cyclin B characteristic of prolonged mitosis, and this is overcome by mutation in *B55^twins^*. Future work will be required to tease out other roles of the Gwl–Endos pathway at this stage of development. Our present findings point to a role for Gwl–Endos in regulating translation and thereby pave the way for future studies to investigate how these mechanisms integrate with pathways regulated by Png. Together, it appears that the Png and Gwl kinases control the timing of the translational activation, repression and destruction of maternal mRNAs to give timely restraint and fine-tuning of the onset of the nuclear division cycles that initiate fly development.

## 4. Resource availability

### (a) Materials availability

All unique/stable reagents generated in this study are available from David Glover (dmglover@caltech.edu) or Hélène Rangone (helenerangone@gmail.com) without restriction.

### (b) Data and code availability

The published article includes mass spectrometry data generated by immunoprecipitation of Endos, CLIP190, CP190 and Cyclin B. The mass spectrometry proteomics data have been deposited to the ProteomeXchange Consortium via the PRIDE [65] partner repository with the dataset identifier PXD036613.

Python codes used for Me31B::GFP granules analysis are provided as electronic supplementary material.

## 5. Methods

### (a) Fly lines and genetics

All stocks were maintained at 25°C in standard media. Their origin is listed in the key resources table. Wild-type Oregon-R (OrR) flies were used as a reference.

Females heterozygous mutant for *png* and carrying either a mutant allele for *microtubule star* (*mts*) or *twins* (*tws*) or a transgene expressing *endos*, used for the analysis of the mitotic phenotype of *png*, were obtained after two generations crosses. In the first step, *png* mutant females (over balancer) were crossed with males carrying *endos* transgene or *tws* or *mts* mutant allele (over balancer). Male progeny obtained from this cross carrying a *png* allele and the *endos* transgene or the *tws* or *mts* allele were then crossed with *png* females (over balancer). The *png* heterozygous female progeny from these second crosses was used for analysis, and at this stage, 50% of the females carried the *endos* transgene or the *tws* or *mts* allele in a phenotypically indistinguishable way.

### (b) Female fertility analysis

Female fertility was tested by taking single newly eclosed females, adding Oregon-R males and checking daily for the onset of egg laying; that day = day 0. Parents were transferred to fresh food every 3 days for a total of 9 days of egg laying, then discarded (three vials were generated per female); progeny were counted (each vial separately) until eclosion was complete. The progeny hatched during 9 days from the onset of hatching were summed up, and the total progeny from each vial was normalized to the averaged total progeny laid by *polo^1^ gwl^Scant/++^* females set up to 1. At least seven females per genotype were tested; the results present the average normalized progeny per female for each genotype. The proportional rescue or enhancement of *polo^1^ gwl^Scant^* sterility was repeatedly observed in stocks assayed up to 7 years apart.

### (c) Propidium iodide staining

Embryos (0–2 h) were collected, dechorionated and fixed by incubation in heptane/methanol (1:1) for at least 5 min on a rotating wheel. Embryos were transferred to Eppendorf tubes and washed 3 × 5 min with 1 ml methanol. For rehydration, 0.5 ml of methanol was replaced with 0.5 ml of PBS for 15 min, followed by 1 ml of PBS for 15 min. Embryos were incubated for 20 min with a propidium iodide solution (1 ml PBS, 5 µl Triton-100X, 2.5 µl of a 10 mg ml^−1^ stock solution of propidium iodide and 10 µl of a 20 mg ml^−1^ solution of RNase A). After three 5 min washes with PBS containing 0.5% Triton-100X, embryos were mounted on a slide in Vectashield Antifade Mounting media.

### (d) Purification of Endos antibody

The anti-Endos serum (rabbit 7648) was affinity purified against 150 µg of GST-Endos antigen (prepared as in [26]) transferred on a PVDF membrane. Excised PVDF membrane carrying Ponceau stained antigen was washed and blocked with TBS containing 0.2% Tween 20 and 3% BSA. In total, 200 µl of serum was added with 1.8 ml of blocking solution and incubated overnight at 4°C with PVDF membrane fragment having the immobilized antigen. After three 10 min washes with TBS containing 0.2% Tween 20, the antibodies retained on the PVDF fragment were eluted by washing three times for 1 min with 400 µl elution buffer (50 mM glycine–HCl, 500 mM NaCl, 0.5% Tween 20, 100 µg ml^−1^ BSA and 0.1% azide) at pH 2.3 and three times for 1 min with 400 µl of elution buffer at pH 1.9. The eluted fractions, pH 2.3 and pH 1.9, were immediately neutralized by the addition of 80 µl or 85 µl of 1.5 M Tris (pH 8.8), respectively. The presence of antibodies in the resulting fractions was confirmed by SDS–PAGE and Coomassie staining.

### (e) Generation of anti-CLIP-190 antibody

The rabbit anti-CLIP-190 antibody was generated against the 851–1468 amino acid fragment of CLIP-190. A pGEX4T-3 plasmid encoding GST-CLIP-190-cc (kind gift of H. Ohkura [66]) was expressed in *Escherichia coli* BL21 after induction with IPTG for 3 h at 37°C. After sonication of the bacterial pellet, the soluble fraction of proteins was retained, and GST-CLIP-190-cc was purified on Glutathione Sepharose 4B beads according to the manufacturer’s instructions. The CLIP-190-cc fragment was then cleaved from the beads with thrombin protease and used for rabbit immunizations (Harlan, UK). The final bleed from rabbit no. 669 was used for immunoprecipitation and western blots. Antibody specificity was determined by western blot against the immunizing antigen, recombinant CLIP-190 expressed in *Drosophila* cell culture and cultured *Drosophila* cells depleted for endogenous CLIP-190.

### (f) Immunoprecipitation from syncytial embryo extracts

For each immunoprecipitation experiment, 0.2 g of 0–2 h embryos from wild-type OrR females were dechorionated and pestle homogenized on ice in 100 µl homogenization buffer (50 mM HEPES (pH 7.6), 150 mM KCl, 2 mM MgCl_2_, 5 mM DTT, 0.1% NP40, 5% glycerol and protease inhibitors cocktail tablet (Complete, EDTA-Free)). Lysates were clarified by centrifugation for 20 min at 4000 r.p.m. at 4°C. Dechorionated embryos in the ‘Froz’ samples were frozen overnight at −80°C before lysis. The concentration of NP-40 was increased to 1% to the homogenization buffer of ‘Buf’. Samples and PhosSTOP phosphatase inhibitor tablet and 1 µM okadaic acid (potassium salt) were added to the homogenization buffer when indicated.

Each immunoprecipitation was carried out with 10 mg of proteins from the clarified extracts to which was added 100 µg (2 µl) of anti-GFP antibody (for control decoy immunoprecipitation) or 8–18 µg (110 µl) of anti-Endos purified antibody. Additional immunoprecipitations were performed against a panel of proteins to compare with Endos pulldowns: Cyclin B as a protein closely related to Endos in promoting Cdk1 activity, CLIP190 as a widely expressed protein associated with microtubules and CP190 as a chromatin protein also found on centrosomes. They were carried out with 10 mg of proteins from the clarified extracts to which was added a volume containing an estimated 1 mg of one of the anti-Cyclin B, anti-Cp190 or anti-CLIP-190 antibodies. The mixtures were then incubated overnight at 4°C on a rotating wheel in a total volume of 5 ml homogenization buffer, washed and added with 400 µl of Dynabeads-Protein A. After further incubation for 2 h and 15 min at 4°C on a rotating wheel, two washes with homogenization buffer containing glycerol and once without glycerol, two elutions were carried out for 5 min at room temperature on a rotating wheel with 500 µl elution buffer (0.5M NH_4_OH and 0.5 mM EDTA). The two eluates were pooled and centrifuged at 13 000 r.p.m. for 2 min, and the cleared eluates were retained.

After three rounds of desiccation to reduce the sample volumes to around 100 µl, samples were readjusted to 1 ml and aliquots were taken for silver staining (SilverQuest Silver Staining Kit) and western-blot analysis, respectively. The remaining proteins were precipitated with cold acetone at −20°C for 10 min, centrifuged and air-dried.

### (g) Mass spectrometry analysis

Mass spectrometry for protein identification of proteins immunoprecipitated from syncytial embryo extracts (as described above) was a service carried out at the Laboratory of Mass Spectrometry, IBB PAS (Warsaw, Poland). Peptide mixtures were analysed by liquid chromatography coupled to tandem mass spectrometry (LC–MS–MS/MS) using Nano-Acquity (Waters) LC system and Orbitrap Velos mass spectrometer (Thermo Electron Corp., San Jose, CA, USA). Prior to the analysis, proteins were subjected to a standard ‘in-solution digestion’ procedure during which proteins were reduced with 50 TCEP (for 60 min at 60°C) and alkylated with 200 mM MMTS (45 min at room temperature) or reduced with 50 mM DTT (for 60 min at 60°C) and alkylated with 200 mM IAM (for 45 min in the darkness at room temperature), where appropriate. Following reduction/alkylation, proteins were digested overnight with trypsin (sequencing Grade Modified Trypsin—Promega V5111). The peptide mixture was applied to RP-18 precolumn (nanoACQUITY Symmetry® C18—Waters 186003514) using water containing 0.1% TFA as mobile phase and then transferred to a nano-HPLC RP-18 column (nanoACQUITY BEH C18—Waters 186003545) using an acetonitrile gradient (5–35% AcN in 180 min) in the presence of 0.05% formic acid with a flow rate of 250 nl min^−1^. The column outlet was directly coupled to the ion source of the spectrometer working in the regime of data-dependent MS to MS/MS switch.

Acquired raw data were processed by Mascot Distiller followed by Mascot Search Engine (Matrix Science, London, UK, on-site licence) against *Drosophila melanogaster* database restricted. The detailed search parameters, including precursor and product ions mass tolerances, enzyme specificity, number of missed cleavages and modifications, are listed in the individual search result files. Peptides with the Mascot Score exceeding the threshold value corresponding to <5% expectation value, calculated by the Mascot procedure, were considered to be positively identified.

### (h) Protein samples preparation and western-blot analysis

For each genotype, 200–400 embryos were collected every 30 min or 1.5 h to prepare 0–1 h or 0–2 h embryo extracts, respectively. Embryos were processed as detailed above for the syncytial embryo extracts except that 6 µl of one-detergent lysis buffer (50 mM Tris (pH 8), 150 mM NaCl, 1% NP40 and protease inhibitors cocktail tablet (Complete Mini EDTA-Free)) were used per 20 embryos for homogenization. In total, 20 µg of soluble proteins were prepared for the western-blot analysis.

Twenty pairs of ovaries were dissected in 0.7% NaCl solution and kept on dry ice until preparation of the extracts following a similar protocol to embryo extracts (5 µl of one-detergent buffer per pair of ovaries). In total, 25 µg of soluble proteins were processed for western-blot analysis.

Embryo or ovary protein samples were transferred onto PVDF membranes according to standard protocols. Proteins were detected by chemiluminescence on X-ray film with a film processor (Agfa, Curix 60) or by combined detection of chemiluminescence and fluorescence with the Odyssey Fc system (Li-COR). After detection of the protein of interest on X-ray film, membranes were deactivated with 10% acetic acid for 10 min at room temperature, water washed and blocked again for incubation with the anti-tubulin reference antibody.

The following primary antibodies were used: rabbit anti-Endos 1:4000 (purified antibody serum no. 7648), rabbit anti-Cyclin B 1:3000 (no. 271), rabbit anti-Cyclin A 1:3000 (no. 270), rabbit anti-Me31B 1:4000 (generous gift from A. Nakamura [55]), mouse anti-Polo 1:200 (MA294) and mouse anti-αTubulin (DM1A).

### (i) RNA extraction and qPCR

RNA was extracted from 20 pairs of ovaries dissected in PBS or from 150 to 200 0–2 h embryos using the RNeasy Mini Kit. On-column digestion with RNase-free DNase was carried out to eliminate genomic DNA. RNA was resuspended in water, and its concentration was measured with a NanoDrop 2000c Spectrophotometer (Thermo Fisher Scientific). RNA was processed with the *Power* CYBR^®^ Green RNA-to-C_T_^TM^ 1-Step Kit (120 ng of RNA per reaction in a total volume of 12 µl) according to the manufacturer’s instructions. Real-time PCR was performed using an Applied Biosystems StepOnePlus^TM^ system (comparative C_T_ experiments) and the oligonucleotides listed in electronic supplementary material, table S4 (alcohol dehydrogenase was used as a reference).

### (j) Immunofluorescence

For stage 14 oocyte analysis, ovaries were dissected and placed directly into 4% paraformaldehyde in PBS for fixation and incubated on a rotating wheel for 15 min at room temperature. After three washes in PBS containing 2% Triton, ovaries were incubated with Phalloidin (1:250) and DAPI (1 mg ml^−1^ stock used at 1:200) on a rotating wheel for 45 min at room temperature. After three washes in PBS containing 2% Triton, ovaries were mounted on a slide in Vectashield Antifade Mounting media. Pictures were acquired using the Leica SP8 confocal microscope with an air 20×/NA objective.

Embryos were collected, dechorionated, fixed with methanol and rehydrated as described above for propidium iodide staining. They were then blocked in PBS containing 0.5% Triton-100X and 1% BSA for 30 min at room temperature on a rotating wheel and then incubated with rabbit anti-GFP antibody (1:800) for 1 h at room temperature. After three washes in PBS containing 0.5% Triton-100X, the samples were incubated with the secondary goat anti-rabbit A488 antibody for 1 h at room temperature in the dark. Embryos were washed three times in PBS containing 0.5% Triton-100X and transferred onto a slide with Vectashield Antifade Mounting Media with DAPI. Immunofluorescence images were acquired using a Leica SP8 confocal microscope with an oil 63×/NA 1.4 objective. Alternatively, embryos in electronic supplementary material, figure S3, were fixed with a 50:50 paraformaldehyde 4%: heptane solution for 15 min on a shaker and devitellinized in methanol. After three washes in methanol, they were stored at −20°C before immunostaining with the anti-Endos antibody (1:200) and anti-Me31B mouse antibody (1:200) or GFP-booster (1:200; Proteintech, Chromo Tek GFP-Booster Alexa Fluor 488). Images were acquired using a Leica SP5 confocal microscope with a 63×/NA 1.4 oil objective (electronic supplementary material, figure S3B) or an Olympus FV3000 confocal microscope with a 60×/NA 1.4 oil objective (figure 3*a*).

### (k) Quantification and statistical analysis

To analyse the *png^1058^/png^3318^* mitotic phenotype, embryos exhibiting 23 or more propidium iodide stained nuclei (having at least undergone four mitotic cycles even in the event of detection of the remaining meiotic polar bodies) were scored in each experiment and standardized to the OrR reference (the average number of embryos laid by females OrR exhibiting ≥23 nuclei was set to 100). Since 50% of the females heterozygous for mutant *png* alleles also carried the *endos* transgene or the *tws* or *mts* allele in a phenotypically indistinguishable way (see §5a), only half of the scored embryos derived from these females have the possibility to develop further than four rounds of mitosis. The standardized percentages of the embryos exhibiting ≥23 nuclei laid by mothers of these genotypes reflect the expected occurrence of the transgene or mutant *tws* or *mts* allele.

The quantification of band intensity corresponding to protein signal on X-ray films was performed with FIJI software [67]. The intensity of a rectangular region around non-overexposed bands was measured and subtracted by the intensity of a rectangular region for a nearby background area. The intensity of the band for a target protein was then divided by the intensity of the band of its corresponding tubulin control for signal normalization in the lane. The normalized signal detected in the wild-type OrR sample was set to 1 and the normalized signal detected in the other lanes (other genetic backgrounds) was calculated as fold change relative to 1.

When protein signal was detected with the Li-COR Odyssey Fc system, quantifications of band intensity were performed according to the manufacturer’s instructions.

To analyse Me31B::GFP granules, images of embryos or ovaries were deconvolved with Huygens software. Processed images were flattened (maximum intensity projection) and a 40 × 40 µm^2^ area representative of the Me31B::GFP staining in the embryo or the oocyte was selected. Embryos were checked for the presence of nuclei (usually scattered DNA in the case of embryos from *endos* mutant mothers), and stage 14 oocytes only were analysed from fixed ovary preparations (oocytes presenting elongated appendages and no nurse cells nuclei with DAPI staining). Representative areas of embryos or oocytes were analysed with a script prepared by Richard Butler (laboratory of Alex Sossick, Gurdon Institute, Cambridge, UK) to measure the area and the fluorescence intensity of the Me31B::GFP signal.

Statistical parameters (value of *n*, mean, s.e.m. and *p*-value) and statistical tests used in individual experiments are reported in the caption of each figure.

## Data Availability

The published article includes mass spectrometry data generated by immunoprecipitation of Endos, CLIP190, CP190 and Cyclin B. The mass spectrometry proteomics data have been deposited to the ProteomeXchange Consortium via the PRIDE [65] partner repository with the dataset identifier PXD036613. Python codes used for Me31B::GFP granules analysis are provided as electronic supplementary material [68].

## References

[1] Tadros W , Lipshitz HD . 2009 The maternal-to-zygotic transition: a play in two acts. Development **136** , 3033–3042. (10.1242/dev.033183) 19700615

[2] Despic V , Neugebauer KM . 2018 RNA tales: how embryos read and discard messages from mom. J. Cell. Sci. **131** , jcs201996. (10.1242/jcs.201996) 29467249

[3] Hamm DC , Harrison MM . 2018 Regulatory principles governing the maternal-to-zygotic transition: insights from Drosophila melanogaster. Open Biol. **8** , 180183. (10.1098/rsob.180183) 30977698 PMC6303782

[4] Vastenhouw NL , Cao WX , Lipshitz HD . 2019 The maternal-to-zygotic transition revisited. Development **146** , dev161471. (10.1242/dev.161471) 31189646

[5] Laver JD , Marsolais AJ , Smibert CA , Lipshitz HD . 2015 Regulation and function of maternal gene products during the maternal-to-zygotic transition in Drosophila. Curr. Top. Dev. Biol. **113** , 43–84. (10.1016/bs.ctdb.2015.06.007) 26358870

[6] Fenger DD , Carminati JL , Burney-Sigman DL , Kashevsky H , Dines JL , Elfring LK , Orr-Weaver TL . 2000 PAN GU: a protein kinase that inhibits S phase and promotes mitosis in early Drosophila development. Development **127** , 4763–4774. (10.1242/dev.127.22.4763) 11044392

[7] Lee LA , Elfring LK , Bosco G , Orr-Weaver TL . 2001 A genetic screen for suppressors and enhancers of the Drosophila PAN GU cell cycle kinase identifies cyclin B as a target. Genetics **158** , 1545–1556. (10.1093/genetics/158.4.1545) 11514446 PMC1461742

[8] Lee LA , Van Hoewyk D , Orr-Weaver TL . 2003 The Drosophila cell cycle kinase PAN GU forms an active complex with PLUTONIUM and GNU to regulate embryonic divisions. Genes Dev. **17** , 2979–2991. (10.1101/gad.1132603) 14665672 PMC289155

[9] Tadros W , Houston SA , Bashirullah A , Cooperstock RL , Semotok JL , Reed BH , Lipshitz HD . 2003 Regulation of maternal transcript destabilization during egg activation in Drosophila. Genetics **164** , 989–1001. (10.1093/genetics/164.3.989) 12871909 PMC1462612

[10] Vardy L , Orr-Weaver TL . 2007 The Drosophila PNG kinase complex regulates the translation of cyclin B. Dev. Cell **12** , 157–166. (10.1016/j.devcel.2006.10.017) 17199048

[11] Tadros W *et al* . 2007 SMAUG is a major regulator of maternal mRNA destabilization in Drosophila and its translation is activated by the PAN GU kinase. Dev. Cell **12** , 143–155. (10.1016/j.devcel.2006.10.005) 17199047

[12] Kronja I , Yuan B , Eichhorn SW , Dzeyk K , Krijgsveld J , Bartel DP , Orr-Weaver TL . 2014 Widespread changes in the posttranscriptional landscape at the Drosophila oocyte-to-embryo transition. Cell Rep. **7** , 1495–1508, (10.1016/j.celrep.2014.05.002) 24882012 PMC4143395

[13] Hara M , Petrova B , Orr-Weaver TL . 2017 Control of PNG kinase, a key regulator of mRNA translation, is coupled to meiosis completion at egg activation. Elife **6** , e22219. (10.7554/eLife.22219) 28555567 PMC5449181

[14] Dorée M , Hunt T . 2002 From Cdc2 to Cdk1: when did the cell cycle kinase join its cyclin partner? J. Cell. Sci. **115** , 2461–2464. (10.1242/jcs.115.12.2461) 12045216

[15] Kishimoto T . 2015 Entry into mitosis: a solution to the decades-long enigma of MPF. Chromosoma **124** , 417–428. (10.1007/s00412-015-0508-y) 25712366 PMC4666901

[16] Zitouni S , Nabais C , Jana SC , Guerrero A , Bettencourt-Dias M . 2014 Polo-like kinases: structural variations lead to multiple functions. Nat. Rev. Mol. Cell Biol. **15** , 433–452. (10.1038/nrm3819) 24954208

[17] Pintard L , Archambault V . 2018 A unified view of spatio-temporal control of mitotic entry: polo kinase as the key. Open Biol. **8** , 180114. (10.1098/rsob.180114) 30135239 PMC6119860

[18] Yu J , Fleming SL , Williams B , Williams EV , Li Z , Somma P , Rieder CL , Goldberg ML . 2004 Greatwall kinase: a nuclear protein required for proper chromosome condensation and mitotic progression in Drosophila. J. Cell Biol. **164** , 487–492. (10.1083/jcb.200310059) 14970188 PMC2171981

[19] Vigneron S , Brioudes E , Burgess A , Labbé JC , Lorca T , Castro A . 2009 Greatwall maintains mitosis through regulation of PP2A. EMBO J. **28** , 2786–2793. (10.1038/emboj.2009.228) 19680222 PMC2750022

[20] Castilho PV , Williams BC , Mochida S , Zhao Y , Goldberg ML . 2009 The M phase kinase Greatwall (Gwl) promotes inactivation of PP2A/B55delta, a phosphatase directed against CDK phosphosites. Mol. Biol. Cell **20** , 4777–4789. (10.1091/mbc.e09-07-0643) 19793917 PMC2777107

[21] Burgess A , Vigneron S , Brioudes E , Labbé JC , Lorca T , Castro A . 2010 Loss of human greatwall results in G2 arrest and multiple mitotic defects due to deregulation of the cyclin B-Cdc2/PP2A balance. Proc. Natl Acad. Sci. USA **107** , 12564–12569. (10.1073/pnas.0914191107) 20538976 PMC2906566

[22] Hara M , Abe Y , Tanaka T , Yamamoto T , Okumura E , Kishimoto T . 2012 Greatwall kinase and cyclin B-Cdk1 are both critical constituents of M-phase-promoting factor. Nat. Commun. **3** , 1059. (10.1038/ncomms2062) 22968705 PMC3658099

[23] Álvarez-Fernández M *et al* . 2013 Greatwall is essential to prevent mitotic collapse after nuclear envelope breakdown in mammals. Proc. Natl Acad. Sci. USA **110** , 17374–17379. (10.1073/pnas.1310745110) 24101512 PMC3808628

[24] Gharbi-Ayachi A , Labbé JC , Burgess A , Vigneron S , Strub JM , Brioudes E , Van-Dorsselaer A , Castro A , Lorca T . 2010 The substrate of greatwall kinase, Arpp19, controls mitosis by inhibiting protein phosphatase 2A. Science **330** , 1673–1677. (10.1126/science.1197048) 21164014

[25] Mochida S , Maslen SL , Skehel M , Hunt T . 2010 Greatwall phosphorylates an inhibitor of protein phosphatase 2A that is essential for mitosis. Science **330** , 1670–1673. (10.1126/science.1195689) 21164013

[26] Rangone H *et al* . 2011 Suppression of scant identifies Endos as a substrate of greatwall kinase and a negative regulator of protein phosphatase 2A in mitosis. PLoS Genet. **7** , e1002225. (10.1371/journal.pgen.1002225) 21852956 PMC3154957

[27] Kim MY *et al* . 2012 Bypassing the greatwall-endosulfine pathway: plasticity of a pivotal cell-cycle regulatory module in Drosophila melanogaster and Caenorhabditis elegans. Genetics **191** , 1181–1197. (10.1534/genetics.112.140574) 22649080 PMC3416000

[28] Glover DM . 2012 The overlooked greatwall: a new perspective on mitotic control. Open Biol. **2** , 120023. (10.1098/rsob.120023) 22754657 PMC3382961

[29] Juanes MA , Khoueiry R , Kupka T , Castro A , Mudrak I , Ogris E , Lorca T , Piatti S . 2013 Budding yeast greatwall and endosulfines control activity and spatial regulation of PP2A(Cdc55) for timely mitotic progression. PLoS Genet. **9** , e1003575. (10.1371/journal.pgen.1003575) 23861665 PMC3701715

[30] Cundell MJ , Bastos RN , Zhang T , Holder J , Gruneberg U , Novak B , Barr FA . 2013 The BEG (PP2A-B55/ENSA/Greatwall) pathway ensures cytokinesis follows chromosome separation. Mol. Cell **52** , 393–405, (10.1016/j.molcel.2013.09.005) 24120663 PMC3898901

[31] Sarkar S , Dalgaard JZ , Millar JBA , Arumugam P . 2014 The Rim15-endosulfine-PP2ACdc55 signalling module regulates entry into gametogenesis and quiescence via distinct mechanisms in budding yeast. PLoS Genet. **10** , e1004456. (10.1371/journal.pgen.1004456) 24968058 PMC4072559

[32] White-Cooper H , Carmena M , Gonzalez C , Glover DM . 1996 Mutations in new cell cycle genes that fail to complement a multiply mutant third chromosome of Drosophila. Genetics **144** , 1097–1111. (10.1534/genetics.112.1097.test) 8913753

[33] Archambault V , Zhao X , White-Cooper H , Carpenter ATC , Glover DM . 2007 Mutations in Drosophila greatwall/Scant reveal its roles in mitosis and meiosis and interdependence with polo kinase. PLoS Genet. **3** , e200. (10.1371/journal.pgen.0030200) 17997611 PMC2065886

[34] Wang P , Pinson X , Archambault V . 2011 PP2A-twins is antagonized by greatwall and collaborates with polo for cell cycle progression and centrosome attachment to nuclei in drosophila embryos. PLoS Genet. **7** , e1002227. (10.1371/journal.pgen.1002227) 21852958 PMC3154958

[35] Von Stetina JR , Tranguch S , Dey SK , Lee LA , Cha B , Drummond-Barbosa D . 2008 α-Endosulfine is a conserved protein required for oocyte meiotic maturation in Drosophila . Development **135** , 3697–3706. (10.1242/dev.025114) 18927152 PMC2654389

[36] Freeman M , Nüsslein-Volhard C , Glover DM . 1986 The dissociation of nuclear and centrosomal division in gnu, a mutation causing giant nuclei in Drosophila. Cell **46** , 457–468. (10.1016/0092-8674(86)90666-5) 3089628

[37] Freeman M , Glover DM . 1987 The gnu mutation of Drosophila causes inappropriate DNA synthesis in unfertilized and fertilized eggs. Genes Dev. **1** , 924–930. (10.1101/gad.1.9.924) 12465624

[38] Shamanski FL , Orr-Weaver TL . 1991 The drosophila plutonium and pan gu genes regulate entry into S phase at fertilization. Cell **66** , 1289–1300. (10.1016/0092-8674(91)90050-9) 1913810

[39] Eichhorn SW , Subtelny AO , Kronja I , Kwasnieski JC , Orr-Weaver TL , Bartel DP . 2016 mRNA poly(A)-tail changes specified by deadenylation broadly reshape translation in drosophila oocytes and early embryos. Elife **5** , e16955. (10.7554/eLife.16955) 27474798 PMC4988829

[40] Hara M , Lourido S , Petrova B , Lou HJ , Von Stetina JR , Kashevsky H , Turk BE , Orr-Weaver TL . 2018 Identification of PNG kinase substrates uncovers interactions with the translational repressor TRAL in the oocyte-to-embryo transition. Elife **7** , e33150. (10.7554/eLife.33150) 29480805 PMC5826265

[41] Nakamura A , Sato K , Hanyu-Nakamura K . 2004 Drosophila cup is an eIF4E binding protein that associates with Bruno and regulates oskar mRNA translation in oogenesis. Dev. Cell **6** , 69–78. (10.1016/s1534-5807(03)00400-3) 14723848

[42] Tritschler F , Eulalio A , Helms S , Schmidt S , Coles M , Weichenrieder O , Izaurralde E , Truffault V . 2008 Similar modes of interaction enable Trailer Hitch and EDC3 to associate with DCP1 and Me31B in distinct protein complexes. Mol. Cell. Biol. **28** , 6695–6708. (10.1128/MCB.00759-08) 18765641 PMC2573232

[43] Wang M , Ly M , Lugowski A , Laver JD , Lipshitz HD , Smibert CA , Rissland OS . 2017 ME31B globally represses maternal mRNAs by two distinct mechanisms during the Drosophila maternal-to-zygotic transition. Elife **6** , e27891. (10.7554/eLife.27891) 28875934 PMC5779226

[44] Wilhelm JE , Hilton M , Amos Q , Henzel WJ . 2003 Cup is an eIF4E binding protein required for both the translational repression of oskar and the recruitment of Barentsz. J. Cell Biol. **163** , 1197–1204. (10.1083/jcb.200309088) 14691132 PMC2173729

[45] Nelson MR , Leidal AM , Smibert CA . 2004 Drosophila Cup is an eIF4E-binding protein that functions in Smaug-mediated translational repression. EMBO J. **23** , 150–159. (10.1038/sj.emboj.7600026) 14685270 PMC1271664

[46] Kinkelin K , Veith K , Grünwald M , Bono F . 2012 Crystal structure of a minimal eIF4E-Cup complex reveals a general mechanism of eIF4E regulation in translational repression. RNA **18** , 1624–1634. (10.1261/rna.033639.112) 22832024 PMC3425778

[47] Satterfield TF , Pallanck LJ . 2006 Ataxin-2 and its Drosophila homolog, ATX2, physically assemble with polyribosomes. Hum. Mol. Genet. **15** , 2523–2532. (10.1093/hmg/ddl173) 16835262

[48] Zhang Y , Ling J , Yuan C , Dubruille R , Emery P . 2013 A role for Drosophila ATX2 in activation of PER translation and circadian behavior. Science **340** , 879–882. (10.1126/science.1234746) 23687048 PMC4078874

[49] Lee J , Yoo E , Lee H , Park K , Hur JH , Lim C . 2017 LSM12 and ME31B/DDX6 define distinct modes of posttranscriptional regulation by ATAXIN-2 protein complex in drosophila circadian pacemaker neurons. Mol. Cell **66** , 129–140.(10.1016/j.molcel.2017.03.004) 28388438

[50] Blagden SP , Gatt MK , Archambault V , Lada K , Ichihara K , Lilley KS , Inoue YH , Glover DM . 2009 Drosophila Larp associates with poly(A)-binding protein and is required for male fertility and syncytial embryo development. Dev. Biol. **334** , 186–197. (10.1016/j.ydbio.2009.07.016) 19631203

[51] Weil TT *et al* . 2012 Drosophila patterning is established by differential association of mRNAs with P bodies. Nat. Cell Biol. **14** , 1305–1313. (10.1038/ncb2627) 23178881 PMC4066581

[52] Sankaranarayanan M , Weil TT . 2020 Granule regulation by phase separation during Drosophila oogenesis. Emerg. Top. Life Sci. **4** , 343–352. (10.1042/ETLS20190155) 32573699 PMC7733668

[53] Davidson A , Parton RM , Rabouille C , Weil TT , Davis I . 2016 Localized translation of gurken/TGF-α mRNA during Axis specification is controlled by access to Orb/CPEB on processing bodies. Cell Rep. **14** , 2451–2462, (10.1016/j.celrep.2016.02.038) 26947065 PMC4823467

[54] Talarek N *et al* . 2010 Initiation of the TORC1-regulated G0 program requires Igo1/2, which license specific mRNAs to evade degradation via the 5’-3’ mRNA decay pathway. Mol. Cell **38** , 345–355. (10.1016/j.molcel.2010.02.039) 20471941 PMC2919320

[55] Nakamura A , Amikura R , Hanyu K , Kobayashi S . 2001 Me31B silences translation of oocyte-localizing RNAs through the formation of cytoplasmic RNP complex during Drosophila oogenesis. Development **128** , 3233–3242. (10.1242/dev.128.17.3233) 11546740

[56] Wilhelm JE , Buszczak M , Sayles S . 2005 Efficient protein trafficking requires trailer hitch, a component of a ribonucleoprotein complex localized to the ER in Drosophila. Dev. Cell **9** , 675–685. (10.1016/j.devcel.2005.09.015) 16256742

[57] Haas G , Braun JE , Igreja C , Tritschler F , Nishihara T , Izaurralde E . 2010 HPat provides a link between deadenylation and decapping in metazoa. J. Cell Biol. **189** , 289–302. (10.1083/jcb.200910141) 20404111 PMC2856893

[58] Liu L , Qi H , Wang J , Lin H . 2011 PAPI, a novel TUDOR-domain protein, complexes with AGO3, ME31B and TRAL in the nuage to silence transposition. Development **138** , 1863–1873. (10.1242/dev.059287) 21447556 PMC3074456

[59] Götze M *et al* . 2017 Translational repression of the Drosophila nanos mRNA involves the RNA helicase Belle and RNA coating by Me31B and Trailer hitch. RNA **23** , 1552–1568. (10.1261/rna.062208.117) 28701521 PMC5602113

[60] Von Stetina JR , Lafever KS , Rubin M , Drummond-Barbosa D . 2011 A genetic screen for dominant enhancers of the cell-cycle regulator α-endosulfine identifies matrimony as a strong functional interactor in Drosophila. G3 Genes Genomes Genet. **1** , 607–613. (10.1534/g3.111.001438) PMC3276179 22384372

[61] Chen L *et al* . 2014 Global regulation of mRNA translation and stability in the early Drosophila embryo by the Smaug RNA-binding protein. Genome Biol. **15** , R4. (10.1186/gb-2014-15-1-r4) 24393533 PMC4053848

[62] Avilés-Pagán EE , Hara M , Orr-Weaver TL . 2021 The GNU subunit of PNG kinase, the developmental regulator of mRNA translation, binds BIC-C to localize to RNP granules. Elife **10** , e67294. (10.7554/eLife.67294) 34250903 PMC8313231

[63] Larouche M *et al* . 2021 Spatiotemporal coordination of Greatwall-Endos-PP2A promotes mitotic progression. J. Cell Biol. **220** , e202008145. (10.1083/jcb.202008145) 33836042 PMC8042607

[64] Sankaranarayanan M , Emenecker RJ , Wilby EL , Jahnel M , Trussina I , Wayland M , Alberti S , Holehouse AS , Weil TT . 2021 Adaptable P body physical states differentially regulate bicoid mRNA storage during early Drosophila development. Dev. Cell **56** , 2886–2901.(10.1016/j.devcel.2021.09.021) 34655524 PMC8555633

[65] Perez-Riverol Y *et al* . 2022 The PRIDE database resources in 2022: a hub for mass spectrometry-based proteomics evidences. Nucleic Acids Res. **50** , D543–D552. (10.1093/nar/gkab1038) 34723319 PMC8728295

[66] Dzhindzhev NS , Rogers SL , Vale RD , Ohkura H . 2005 Distinct mechanisms govern the localisation of Drosophila CLIP-190 to unattached kinetochores and microtubule plus-ends . J. Cell. Sci. **118** , 3781–3790. (10.1242/jcs.02504) 16105886

[67] Schindelin J *et al* . 2012 Fiji: an open-source platform for biological-image analysis. Nat. Methods **9** , 676–682. (10.1038/nmeth.2019) 22743772 PMC3855844

[68] Rangone H , Bond L , Weil T , Glover DM . 2024 Supplementary material from: Greatwall-Endos-PP2A/B55Twins network regulates translation and stability of maternal transcripts in the Drosophila oocyte-to-embryo transition. Figshare. (10.6084/m9.figshare.c.7262706) PMC11286125 38896085

